# Propagule-Type Specificity in Arbuscular Mycorrhizal Fungal Communities in Early Growth of *Allium tuberosum*

**DOI:** 10.3390/microorganisms13061430

**Published:** 2025-06-19

**Authors:** Irem Arslan, Kohei Takahashi, Naoki Harada, Kazuki Suzuki

**Affiliations:** 1 Graduate School of Science and Technology, Niigata University, 8050 Ikarashi-2, Nishi-ku, Niigata 950-2181, Japan; 2 Institute of Science and Technology, Niigata University, 8050 Ikarashi-2, Nishi-ku, Niigata 950-2181, Japan

**Keywords:** arbuscular mycorrhizal fungi, high-throughput sequencing, propagule-specific AMF distribution, life-history strategy

## Abstract

Arbuscular mycorrhizal fungi (AMF) exhibit diverse strategies for colonization and survival, yet the extent to which different propagule types—roots, extraradical hyphae, and spores—contribute to these processes remains unclear. In a pot experiment using *Allium tuberosum* and soils from three field sites, we characterized AMF communities in root, hyphal, and spore fractions through 18S rRNA gene sequencing. A total of 427 OTUs were identified, with *Glomus* and *Paraglomus* dominating. Root fractions contained significantly more OTUs than hyphal fractions, suggesting strong specialization for intraradical colonization. Only a small subset of taxa occurred across all propagule types. Indicator species analysis revealed 21 OTUs with significant associations, mainly in root and hyphal fractions, while spore-specific taxa were rare. PERMANOVA revealed that both propagule type and soil type shaped the community structure, with propagule identity being the stronger factor. These results highlight propagule-type specialization as a key ecological trait in AMF and underscore the importance of examining multiple fungal compartments to fully capture AMF diversity and function.

## 1. Introduction

Arbuscular mycorrhizal fungi (AMF) are soil-borne microscopic fungi that establish mutualistic relationships with approximately 80% of terrestrial plants. In these symbiotic associations, AMF capture carbon from the host plant derived from photosynthesis, while delivering nutrients that are difficult to obtain from the soil, especially phosphorus, to the plant [1,2]. AMF also enhance plant tolerance to environmental stressors such as drought, salinity, and pathogen pressure [3,4,5]. In addition, AMF contribute to soil aggregation and structural stability through their extensive hyphal networks and glomalin secretion, thereby supporting broader ecosystem functions such as carbon sequestration and nutrient cycling [6,7,8].

AMF exist in three primary morphological forms during their life cycle: spores, extraradical hyphae, and intraradical hyphae [9]. Each propagule type serves a distinct ecological function, as follows: arbuscules facilitate active nutrient exchange between the host plant and the fungus, extraradical hyphae facilitate soil nutrient acquisition, and spores ensure fungus survival and reproduction [10,11]. While the majority of AMF diversity research has relied on morphological identification of spores or microscopy of colonized roots, DNA metabarcoding has become common in the study of AMF communities in roots and soils with the expansion of molecular techniques [12,13]. Varela-Cervero et al. [14,15] demonstrated propagule-specific distributions in AMF taxa, with only partial overlap between root, spore, and hyphal fractions. Likewise, Hart and Klironomos [16] provided evidence for functional differences among propagule types by comparing colonization success from various inoculum sources. These findings imply that AMF taxa may employ diverse survival strategies shaped by ecological trade-offs between intraradical symbiosis, reproductive effort, and soil exploitation. This underscores the importance of investigating all three propagule types in order to fully capture their functional and taxonomic diversity. Therefore, to accurately capture the functional and taxonomic diversity of AMF communities, it is essential to investigate all three propagule types. Studies that omit spores and hyphae may substantially underestimate AMF diversity in natural systems.

Comparative research has also revealed notable differences between root-associated and extraradical hyphal AMF diversity. Root-associated communities tend to exhibit greater diversity due to their close association with individual host plants and root traits such as root diameter and plant developmental stage, which influence AMF richness and community composition [17,18]. In contrast, hyphal communities typically show lower richness, with diversity being more strongly influenced by microbial interactions within the soil matrix and the composition of root-derived exudates [19,20]. Factors such as host plant identity, soil nutrient dynamics, and environmental disturbances play key roles in shaping these differences between root and hyphal AMF communities [21,22,23]. Among these, soil nutrient availability and disturbance regimes are particularly influential in structuring AMF community composition and propagule distribution patterns [24,25,26]. Moreover, several AMF families, including Gigasporaceae and Glomeraceae, display varying sensitivities to soil texture, which affects their colonization strategies, sporulation, and hyphal growth patterns [27,28]. However, it remains poorly understood how propagule-specific AMF communities are influenced by inoculum sources with contrasting edaphic histories, particularly under standardized environmental conditions in controlled experiments.

*Allium* species—including *Allium tuberosum*—are widely considered as suitable model hosts for mycorrhizal studies, as their ease of cultivation and their weakly branched roots confer a strong dependence on mycorrhizal fungi for nutrient uptake [29,30]. Leveraging this host, we aimed to assess the diversity, community structure, and ecological strategies of AMF in three main propagule types: intraradical structures, extraradical hyphae in soil, and spores. Specifically, we investigated how AMF community composition varies among root, hyphal, and spore fractions, and whether certain AMF taxa exhibit propagule-type preferences indicative of functional specialization. To address these objectives, we conducted a controlled pot experiment using *Allium tuberosum* as a host plant inoculated with AMF originating from three contrasting soils: a well-drained, nutrient-poor sandy regosol; an organic-matter-rich andosol with high phosphorus-fixing capacity; and a gleysol formed under water-saturated, reduced conditions. High-throughput amplicon sequencing of the 18S rRNA gene was utilized to characterize the taxonomic composition of AMF communities across different propagule types, providing new insights into the life-history traits and survival strategies of AMF taxa.

## 2. Materials and Methods

### 2.1. Soil Sources for AMF Inocula

Three ecologically distinct topsoil samples (0–15 cm depth) were collected from Niigata Prefecture, Japan, to serve as sources of AMF inocula. Sandy regosol soil was obtained on 25 April 2019 from a horticultural field located on the Ikarashi Campus of Niigata University (37°52′02.9″ N, 138°56′29.4″ E). Gleysol soil was sampled on 26 October 2023 from a drained paddy field at the Shindori Station (37°51′21.8″ N, 138°57′37.0″ E), part of the Field Center for Sustainable Agriculture and Forestry at Niigata University. On the same day, andosol soil was collected from a forested area at the Muramatsu Station (37°41′22.7″ N, 139°11′42.0″ E), which is affiliated with the university’s Main Farm and Nursery. After field sampling, each soil type was gently homogenized, passed through a 2 mm stainless-steel sieve, and all visible roots and plant residues were manually removed before the soil samples were sealed and stored at 4 °C. The stored soil samples were used as AMF inocula within 2 months. The soil pH (H_2_O, 1:2.5 *w*/*v*) was 5.87 for the sandy regosol, 4.83 for the gleysol, and 3.94 for the andosol.

### 2.2. Soil Preparation and Experimental Setup

The base soil mixture consisted of a 1:1 (*v*/*v*) sterilized combination of river sand and fine-grained red bead soil (akadama), both sieved to <1 mm and autoclaved twice at 121 °C for 20 min at 24 h intervals. To prepare the experimental soil, the AMF inoculum was incorporated at a 1:9 (*w*/*w*) ratio into the sterilized base mixture. A standardized fertilization regime was applied by supplementing the soil with NH_4_NO_3_ and KH_2_PO_4_ to achieve a target N:P:K ratio of 30:20:25 kg/ha. This uniform, nutrient-defined soil mixture ensured that any subsequent differences in AMF community assembly could be attributed solely to the origin of the inoculum, rather than to variability in texture, pH, or nutrient status among the source soils. A schematic overview of the experimental workflow is provided in Supplementary Figure S1.

### 2.3. Seed Sterilization, Germination, and Plant Cultivation

Garlic chives (*Allium tuberosum*), a perennial species in the family Amaryllidaceae, were used as the host plant. Seeds were surface-sterilized in a 1% sodium hypochlorite solution for 10 min, followed by three rinses with sterile deionized water. Germination was carried out in sterile Petri dishes lined with filter paper for one week. Uniform seedlings were then transplanted into 27 pots, with three biological replicates per inoculum source and six seedlings per pot. The pots were maintained in a growth chamber under controlled conditions (25 °C day/23 °C night; 12 h light/12 h dark; relative humidity 60%) with internal air circulation. Soil moisture was maintained at 60% of the maximum water-holding capacity using sterile deionized water. A destructive sampling protocol was employed to ensure independent biological replicates. Samples were collected at three time points of 2, 4, and 6 weeks after transplantation.

### 2.4. DNA Extraction from Root Samples

The roots of garlic chives were separated from the shoots using sterile scissors following a 15 s ultrasonication treatment to remove adherent soil particles. Fine root segments were excised, flash-frozen in liquid nitrogen, and ground to a fine powder using a mortar and pestle. Genomic DNA was extracted from 20 mg of root tissue using the ISOPLANT DNA extraction kit (Nippon Gene, Tokyo, Japan), according to the manufacturer’s instructions. Extracted DNA samples were stored at −20 °C until further analysis.

### 2.5. DNA Extraction from Hyphae and Spore Fractions

AMF spores and extraradical hyphae were extracted from 50 g of soil using a modified wet sieving and sucrose gradient centrifugation method adapted from Varela-Cervero et al. [14]. The spores were separated via 15 s of ultrasonication treatment and two-step sucrose layering (20–60%), followed by overnight incubation and manual cleaning under a microscope. The remaining residues were used for hyphal isolation. Both fractions were stored at −20 °C for DNA extraction.

DNA extraction from the spore fractions was performed using a protocol adapted from the Instagene Matrix method (Bio-Rad, Hercules, CA, USA). A volume of 200 μL Instagene solution was added to the purified spore suspension in a 1.5 mL microcentrifuge tube. The spores were homogenized using a sterile micropestle, followed by incubation at 56 °C for 30 min and subsequently at 95 °C for 10 min. The samples were then centrifuged at 13,000 rpm for 5 min, and the resulting supernatant was collected and stored at −20 °C for molecular analysis.

DNA was extracted from purified extraradical hyphae using the ISOPLANT DNA extraction kit (Nippon Gene, Tokyo, Japan), following the manufacturer’s instructions. The extracted DNA samples were stored at −20 °C until PCR amplification.

### 2.6. PCR Amplification and Sequencing

The universal primer pair AMV4.5NF and AMDGR [31], which comprised overhang adapter sequences for the Nextera XT index primers (Illumina, San Diego, CA, USA), were used in a polymerase chain reaction (PCR) using the DNA extracts from all samples as templates. A final reaction volume of 25 µL was used for the PCR to amplify the 18S rRNA gene. Briefly, 1 µL of template DNA, 0.1 µL of forward and reverse primers (50 µM, 10P), 11.5 µL of water, and 12.5 µL of Ex Premier DNA Polymerase (Takara Bio, Kusatsu, Japan) made up the PCR reaction mixture. The PCR conditions for amplifying arbuscular mycorrhizal fungi were established as follows: initial denaturation at 95 °C for 3 min, followed by 35 cycles of denaturation at 95 °C for 30 s, annealing at 58 °C for 40 s, and elongation at 72 °C for 40 s, with a final extension at 72 °C for 5 min. Amplicon purification was performed using Agencourt AMPure XP (Beckman Coulter, Brea, CA, USA), following the manufacturer’s instructions.

The index PCR was conducted under the following thermocycling conditions: 10 cycles of denaturation at 95 °C for 30 s, annealing at 55 °C for 30 s, and extension at 72 °C for 30 s, followed by a final extension at 72 °C for 5 min. Amplicons were subsequently purified a second time using the same purification protocol. The quantification of purified amplicons was performed using the Qubit™ Flex Fluorometer (Thermo Fisher Scientific, Waltham, MA, USA) with the Qubit™ dsDNA High Sensitivity (HS) Assay Kit. Equimolar amounts of purified amplicons were pooled, and paired-end sequencing (2 × 300 bp) was performed on the Illumina MiSeq platform using the MiSeq Reagent Kit v3 (Illumina, San Diego, CA, USA). The sequence data have been deposited in the DNA Data Bank of Japan (DDBJ) under the accession number PRJDB20733.

### 2.7. Bioinformatics and Statistical Analysis

Raw sequences were demultiplexed and quality-filtered using QIIME2 (version 2022.11) [32]. DADA2 was utilized for denoising, chimera removal, and the generation of amplicon sequence variants (ASVs) [33]. Representative sequences were taxonomically assigned using the MaarjAM database released in 2021 [13] as a reference through the QIIME2 plugin feature-classifier. Only sequences affiliated with the subphylum Glomeromycotina were retained for downstream analyses. Samples yielding fewer than 5000 Glomeromycotina reads were excluded from diversity and community structure analyses.

Alpha diversity metrics were calculated using the diversity plugin in QIIME2 based on operational taxonomic units (OTUs) clustered at 97% sequence similarity. Beta diversity was assessed using weighted UniFrac distance matrices and visualized via Principal Coordinates Analysis (PCoA). The significance of differences in AMF community composition was evaluated by PERMANOVA (adonis2 function, vegan package in R version 4.4.1), and homogeneity of dispersion was tested using PERMDISP.

Statistical comparisons of alpha diversity among soil types, propagule fractions, and time points were performed using the Kruskal–Wallis test followed by post hoc Dunn tests with Benjamini–Hochberg correction. Indicator species analysis was performed using the indicspecies package (version 1.8.0) [34] to identify OTUs significantly associated with specific propagule types or soil sources. All data visualization and statistical analyses were conducted in RStudio (version 2024.06) using the tidyverse, ggplot2, and vegan packages.

## 3. Results

### 3.1. Overall AMF Taxonomic Diversity

High-throughput sequencing identified a total of 427 OTUs, encompassing 18 genera and 11 families of AMF, and representing 84 species across all propagule fractions. Of these 427 OTUs, only 23 were shared among all three soil types. Sandy regosol exhibited the highest number of unique OTUs (153), followed by gleysol (109) and andosol (76). The overlap and uniqueness of OTU composition among the soil types are illustrated in Supplementary Figure S2, with the corresponding OTU list provided in Supplementary Table S1.

The 20 most abundant OTUs, ranked by total sequence counts, were primarily associated with major AMF families, including Glomeraceae, Gigasporaceae, Acaulosporaceae, and Paraglomeraceae. These dominant OTUs were largely affiliated with well-known and widely distributed genera such as Glomus, Scutellospora, Acaulospora, and Paraglomus. However, several OTUs could not be taxonomically resolved at the genus or species level, suggesting the presence of novel or underrepresented lineages within environmental AMF communities. The high proportion of “unassigned” taxa among the top OTUs highlights the extent of unexplored AMF diversity.

**Figure 1:**
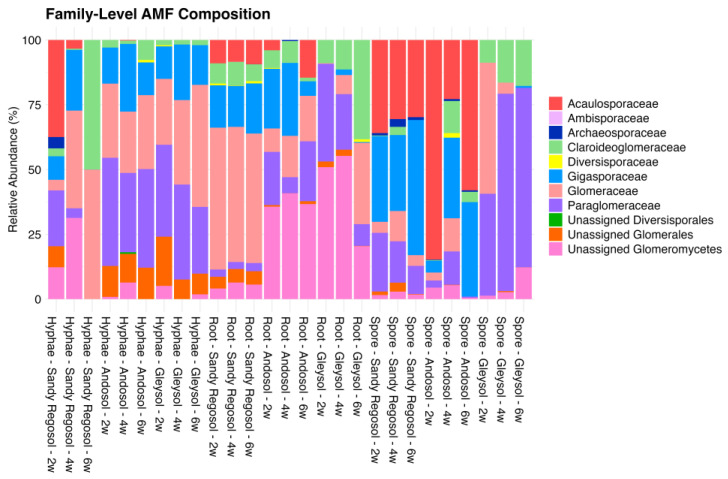
Relative abundance of AMF families identified in hyphae, root, and spore fractions across three soil types (sandy regosol, andosol, and gleysol) and three sampling time points (2, 4, and 6 weeks).

These taxonomic trends were also reflected in the relative abundance profiles at the family level for all samples (Figure 1). AMF communities varied across propagule types, with Glomeraceae showing the highest relative abundance overall, particularly in root and hyphal fractions. In contrast, spore fractions had greater taxonomic heterogeneity with greater proportions of Gigasporaceae, Acaulosporaceae, and Paraglomeraceae, particularly in sandy regosol and andosol samples. Minor changes over sampling weeks were also observed, including a progressive decline of unclassified families, such as unassigned Glomeromycetes, from hyphal compartments over time. Genus-level relative abundance patterns are presented in Supplementary Figure S3. *Glomus* remained consistently dominant in both root and hyphal fractions, whereas spore fractions exhibited more heterogeneous profiles, with prominent representation from *Scutellospora*, *Gigaspora*, and *Acaulospora*.

### 3.2. AMF Diversity Patterns by Propagule Type

**Figure 2:**
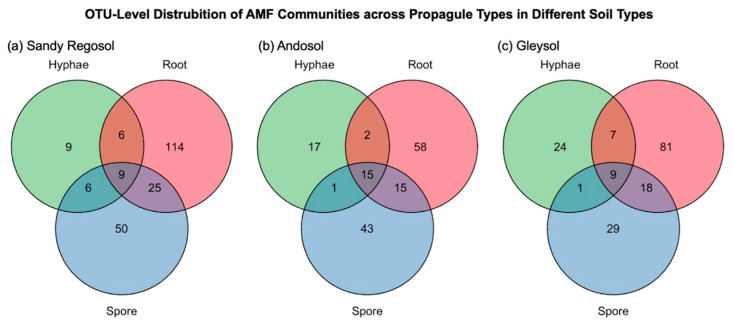
Venn diagrams showing the number of unique and shared AMF OTUs among hyphal, root, and spore fractions in (**a**) sandy regosol, (**b**) andosol, and (**c**) gleysol.

Among the 427 detected OTUs, 34 were shared across all three propagule types, representing core AMF community members capable of producing multiple propagule structures. In contrast, 199 OTUs were unique to the root fraction, 96 to the spore fraction, and 27 to the hyphal fraction, indicating strong compartmentalization of AMF taxa based on propagule type. When examined by soil type, sandy regosol exhibited the highest degree of compartmentalization, with 114 root-specific, 50 spore-specific, and 9 hyphae-specific OTUs, along with 9 OTUs shared across all three propagule types. In andosol, 58 root-specific, 43 spore-specific, and 17 hyphae-specific OTUs were identified, with 15 OTUs shared among all propagule types. Gleysol contained 81 root-specific, 29 spore-specific, and 24 hyphae-specific OTUs, with 9 OTUs common to all fractions. These patterns are visualized in Figure 2, which displays OTU distribution among propagule types separately for each soil source.

Taxonomically, several of the OTUs shared across all propagule types were affiliated with well-characterized genera such as *Scutellospora*, *Acaulospora*, *Paraglomus*, and *Glomus*, belonging to the families Glomeraceae, Gigasporaceae, Acaulosporaceae, and Paraglomeraceae. OTUs unique to each propagule type also exhibited distinct taxonomic patterns. Root-specific OTUs were predominantly assigned to Glomeraceae, while spore-specific OTUs included members of both Acaulosporaceae and Diversisporaceae. In contrast, hyphal-specific OTUs showed limited taxonomic diversity and were primarily affiliated with *Paraglomus* or remained unclassified Glomeromycetes.

### 3.3. Dominant Taxa Show Distinct Propagule Preferences

Among the top 20 OTUs, 10 were most abundant in the hyphal fraction, including *Paraglomus* VTX00348 (OTU003), *Glomus* VTX00219 (OTU004), and several unassigned Glomeromycetes and Glomerales. Six OTUs were dominant in the spore fraction, such as *Acaulospora* VTX00024 (OTU002 and OTU011) and *Scutellospora* VTX00049 (OTU015). The remaining four OTUs showed the highest abundance in the root fraction, including *Glomus* VTX00166 (OTU017) and multiple unassigned Glomeromycetes (e.g., OTU005 and OTU012). Notably, none of the top 20 OTUs were uniformly distributed across all propagule types. Each OTU showed a clear preference for a single dominant compartment, indicating strong structural partitioning within the AMF community.

While the propagule preferences of dominant OTUs were generally consistent, their relative abundances and distribution patterns varied according to the origin of the AMF inoculum. These patterns are examined in greater detail in the following section at the OTU level.

Alpha diversity analysis based on observed richness values showed consistent differences among propagule types across all soils and time points (Supplementary Figure S4). In sandy regosol, Kruskal–Wallis tests revealed significant richness differences among propagule types at all time points (2w: *p* = 0.0273; 4w: *p* = 0.0321; 6w: *p* = 0.0423). Post hoc Dunn tests indicated that richness in root fractions was significantly higher than that found in hyphal fractions at each time point (2w: *p* = 0.0109; 4w: *p* = 0.0132; 6w: *p* = 0.0200). Differences involving the spore fraction were not statistically significant. In andosol and gleysol, similar trends were observed. Root fractions showed significantly higher richness than hyphal fractions at 4 and 6 weeks (andosol 4w: *p* = 0.0249; 6w: *p* = 0.0191; gleysol 4w: *p* = 0.0197; 6w: *p* = 0.0102). A three-way ANOVA revealed significant interaction effects between soil type and propagule type (*p* = 0.0007), and among the soil type, propagule type, and sampling week (*p* = 0.0034). The main effects of soil, propagule type, or week were not significant individually (Supplementary Table S3).

The effects of soil type, propagule type, and sampling week on AMF community composition were evaluated using PERMANOVA based on weighted UniFrac distance matrices. The full model explained 68.0% of the total variation in community structure (R^2^ = 0.68048, F = 4.3412, *p* = 0.001), with 31.9% of the variation remaining unexplained. A term-only PERMANOVA revealed that soil type (R^2^ = 0.17913, *p* = 0.001) and propagule type (R^2^ = 0.14284, *p* = 0.001) were significantly associated with variation in AMF community composition. The sampling week had no significant effect (R^2^ = 0.03830, *p* = 0.096). The interaction between soil type and propagule type was also significant (R^2^ = 0.17821, *p* = 0.001), whereas all other interactions were not statistically significant (Supplementary Table S4). These findings indicate that AMF community composition was primarily shaped by soil origin and propagule type, with only minor contributions from temporal variation over the sampling period.

### 3.4. Propagule Preferences of Dominant AMF OTUs

**Figure 3:**
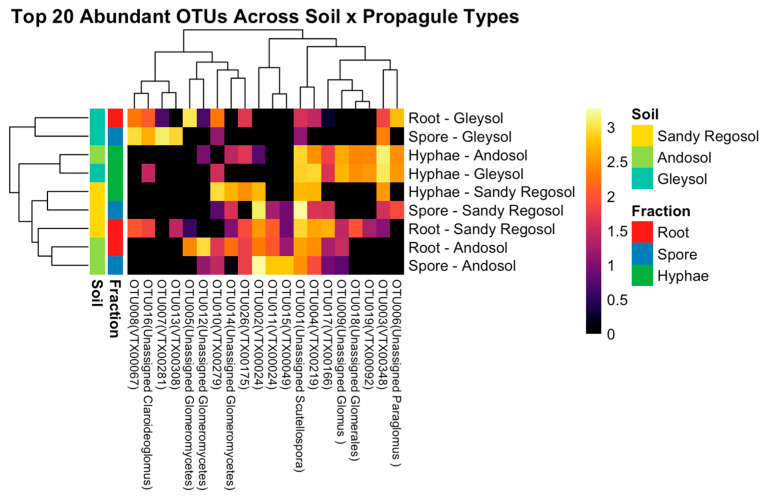
Heatmap of the 20 most abundant AMF OTUs across soil types and propagule fractions. The heatmap illustrates log-transformed relative abundance patterns of the 20 most dominant OTUs in each propagule type (root, spore, hyphae) within three distinct soil types (sandy regosol, andosol, gleysol). Rows represent the combination of soil type and propagule fraction, while columns represent individual OTUs annotated with their genus- or family-level taxonomy. Hierarchical clustering of rows and columns highlights both structural (propagule-based) and edaphic (soil-based) compartmentalization of AMF taxa.

Taxon-specific distribution patterns across propagule types revealed distinct ecological strategies among dominant OTUs. Some taxa were detected in all three fractions, reflecting differences in colonization behavior, dispersal capacity, and environmental adaptability. To visualize these patterns, a clustered heatmap of the 20 most abundant OTUs across all soil types and propagule fractions was generated (Figure 3). The heatmap revealed clear co-occurrence clusters corresponding to propagule- and soil-specific preferences. Several OTUs were exclusively detected in the root fraction and were absent from the hyphal fraction, suggesting specialization for intraradical colonization. For example, unassigned Glomeromycetes (OTU005) were consistently observed in the root fractions of all three soils but were entirely absent from hyphal compartments. Similarly, *Paraglomus* VTX00281 (OTU007) was detected in the root and spore fractions in gleysol, but not in the hyphal fraction, despite its high abundance in spores. In sandy regosol, unassigned *Glomus* (OTU009) and *Glomus* VTX00175 (OTU026) were identified as root-exclusive OTUs.

A small subset of OTUs was detected in all three propagule types in multiple soil types. Unassigned *Scutellospora* (OTU001) was consistently present in the root, hyphae, and spore fractions of all soil types, with particularly high abundance in the spore fraction of sandy regosol. *Paraglomus* VTX00348 (OTU003) was also found in all fractions in both gleysol and sandy regosol. Conversely, we also found that some OTUs were detected in the hyphal fraction and absent from the root fraction. In andosol, *Paraglomus* VTX00348 (OTU003) showed high abundance in the hyphal fraction, while it was not detected in the root and spore fractions. There were similar observations for unassigned *Paraglomus* (OTU006) in andosol and unassigned *Glomus* (OTU009) in gleysol. In addition, *Claroideoglomus* VTX00279 (OTU010) was detected in hyphae and spore fractions in sandy regosol but was absent from the root compartment.

### 3.5. Indicator Taxa Differentiate Propagule Fractions

**Figure 4:**
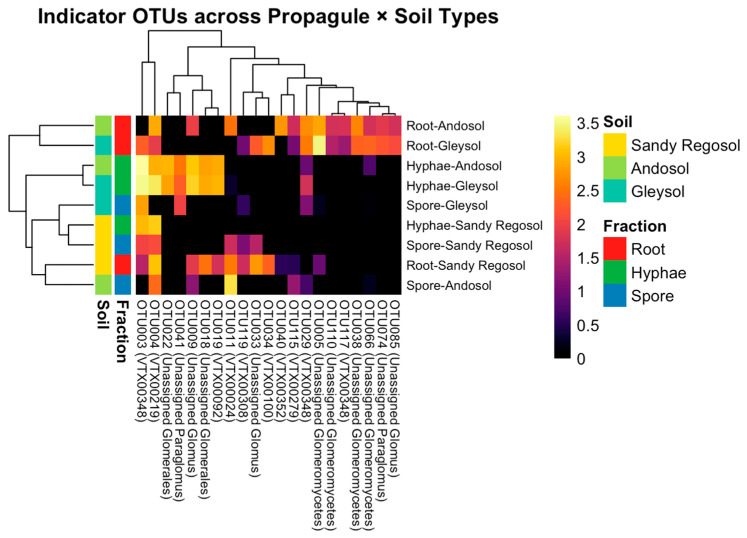
Heatmap showing the distribution of indicator OTUs across different propagule types (root, hyphae, spore) and soil types (andosol, gleysol, sandy regosol). The *y*-axis represents propagule–soil combinations, while the *x*-axis represents individual indicator OTUs identified through IndVal analysis. Cell colors indicate the log10-transformed abundance (log10(count + 1)) of each OTU within each propagule–soil combination. Propagule types and soil types are denoted by side color bars.

Indicator species analysis identified 21 OTUs significantly associated with specific propagule types (*p* < 0.05) (Figure 4; Supplementary Table S5). Of these, 13 OTUs were associated with root fractions, 6 with hyphal fractions, and 2 with combinations of root, hyphal, or spore fractions. Several OTUs previously identified as dominant taxa also emerged as significant indicators. These included OTU005 (unassigned Glomeromycetes), OTU003 (VTX00348), and OTU004 (VTX00219). OTU005 showed a strong preference for root fractions in both andosol and gleysol soils, whereas OTU003 and OTU004 were predominantly associated with hyphal fractions in the same soils.

In contrast, spore fractions supported fewer indicator OTUs and exhibited relatively low overall abundance. A limited number of OTUs, such as OTU041 and OTU074 (both unassigned *Paraglomus*), showed weak but statistically significant associations with spore fractions, particularly in gleysol and sandy regosol soils.

Overall, root and hyphal fractions harbored a greater diversity and an abundance of indicator OTUs compared to spore fractions, and the composition of indicator taxa varied across the soil types.

## 4. Discussion

### 4.1. High AMF Taxonomic Diversity and Propagule-Specific Functional Specialization Characterize Community Assembly

The predominance of *Glomus* and *Paraglomus* in our study is consistent with that of previous reports identifying these genera as common in early successional or disturbed environments [35]. Nevertheless, a substantial proportion of OTUs remained unassigned to any known genus, reflecting the persistent limitations of current AMF reference databases such as MaarjAM [36,37]. These unidentified taxa likely represent cryptic AMF diversity with potential ecological roles adapted to specific microhabitats or host plants. The root fraction consistently exhibited the highest AMF richness compared to hyphal and spore fractions across all soil types, underscoring the critical role of roots as primary sites for AMF colonization and diversity. The functional differentiation among propagule types aligns with our findings that underlined well-distinguished infective capabilities of AMF propagules [38]. Moreover, the greater taxonomic richness observed in root fractions, primarily affiliated with Glomeraceae, suggests that root-associated AMF diversity is strongly influenced by host plant traits such as root diameter and developmental stage. This further highlights the importance of root exudation patterns and root architecture in shaping intraradical AMF communities [17,39,40,41].

The distribution of OTUs across propagule types highlights functional specialization within the AMF community. Only 34 OTUs were shared among all three fractions, suggesting that relatively few generalist taxa are capable of colonizing plant roots, extending extraradical hyphae, and producing spores. In contrast, the large number of OTUs restricted to a single propagule type indicates structural and potentially ecological differentiation in propagule-based survival strategies. Taxonomically, root-exclusive OTUs were primarily affiliated with Glomeraceae, consistent with specialization for intraradical colonization and efficient host resource utilization [38,42]. In comparison, spore-exclusive OTUs were dominated by members of Acaulosporaceae and Diversisporaceae, reflecting a strategy centered on durable spore formation and long-term environmental persistence [1,43].

Hyphae-exclusive OTUs were predominantly assigned to *Paraglomus* and unassigned Glomeromycetes, suggesting a specialization in extraradical proliferation and resource foraging [44,45]. Although some taxa were detected exclusively in the hyphal fraction, it is unlikely that these taxa exist solely as mycelium. Rather, their absence from root compartments likely reflects low colonization density, while their extensive extraradical mycelial networks are more readily detectable in soil. These findings support the notion that AMF structure and ecology are shaped by propagule type. Dominant OTUs exhibited clear compartmental preferences, as follows: *Paraglomus* VTX00348 and *Glomus* VTX00219 were abundant in hyphal fractions; *Acaulospora* VTX00024 and *Scutellospora* VTX00049 were dominant in spore fractions; and root-associated OTUs, such as unassigned Glomeromycetes and *Glomus* VTX00166, showed strong affinity for roots. The absence of uniformly distributed dominant OTUs reinforces the concept of functional differentiation, with each taxon occupying distinct ecological niches defined by the dispersal mode, colonization strategy, or survival capacity.

### 4.2. Inoculum Origin Effect on AMF Community Composition

The strong partitioning of AMF communities among sandy regosol, andosol, and gleysol—particularly the limited number of shared OTUs—emphasizes the influence of inoculum origin in shaping community composition. Despite the uniform base soil mixture used across all treatments, the resulting AMF communities differed markedly depending on the source of the inocula, supporting the idea that legacy effects from local soils shape the initial AMF pools. This outcome is unsurprising given the contrasting land-use histories of the three soils, including a nutrient-poor, intermittently tilled sandy regosol, an organic-matter-rich forest andosol, and a periodically water-logged gleysol formerly under paddy cultivation [46]. Each soil type also harbored characteristic taxa, as follows: the regosol inoculum was dominated by fast-colonizing Glomeraceae OTUs, such as *Glomus* and *Paraglomus*; the andosol favored organic matter specialists in Acaulosporaceae and Paraglomeraceae; whereas the gleysol was enriched in *Paraglomus* and several unassigned Glomeromycetes lineages. This observation aligns with the findings of Prado-Tarango et al. [47], who introduced three ecologically distinct AMF inocula into a sterilized substrate and observed unique colonization patterns in the host grasses. Similarly, Xu et al. [25] and Hazard et al. [48] reported that long-term edaphic history exerts a stronger influence on AMF community structure than short-term environmental conditions during host development. In addition, studies by Lekberg et al. [24] and Balestrini et al. [27] have demonstrated that soil texture significantly influences AMF assemblages, with families such as Glomeraceae and Gigasporaceae showing distinct preferences for clay and sandy soils, respectively. Such adaptations may underline the legacy effects observed in inoculum-derived communities.

Also, consistent with these findings, our PERMANOVA results demonstrated that both the origin of the AMF inoculum and the propagule type exerted significant independent and interactive effects on AMF community composition, whereas temporal variation across sampling weeks had only a minor influence. Collectively, these results emphasize that the composition of the initial inoculum, shaped by long-term environmental history, can exert lasting effects on the AMF community structure, even under standardized growth conditions.

### 4.3. Propagule Preferences Exemplify Functional Specialization and Survival Strategies Among AMF Taxa

The distribution patterns of dominant AMF taxa across different propagule types revealed distinct ecological strategies related to colonization behavior, dispersal mechanisms, and environmental adaptability. Several taxa, including unassigned Glomeromycetes and *Paraglomus* VTX00281, were consistently found in root and spore fractions but were nearly absent in hyphal fractions. This pattern suggests specialization for intraradical colonization and sporulation, with limited investment in extraradical mycelial development. Such root-dominant lifestyles may reflect a greater dependency on host-derived carbon and a prioritization of symbiotic nutrient exchange over soil foraging [1,49]. In contrast, other OTUs—such as *Paraglomus* VTX00348 and unassigned *Paraglomus* in andosol and unassigned *Glomus* in gleysol—were highly abundant in the hyphal fraction while being nearly undetectable in the root and spore fractions. These taxa likely employ a colonization strategy centered on soil foraging via extensive extraradical hyphal networks rather than root colonization. This strategy may confer an advantage in nutrient-poor or spatially heterogeneous environments [42,44,45]. A few generalist taxa, such as unassigned *Scutellospora* and *Paraglomus* VTX00348, were detected across all propagule fractions in multiple soil types. These taxa appear to combine intraradical colonization, soil exploration, and reproductive capacity, suggesting a flexible strategy that enables persistence across diverse environmental conditions. Such generalist behavior may provide resilience by balancing the phases of symbiotic interaction and free-living growth [50,51].

Recent studies have emphasized that AMF survival strategies are strongly influenced by their dispersal capacity and ecological flexibility. Tipton et al. [52] demonstrated that dispersal potential varies among AMF taxa and affects colonization success, particularly in fragmented habitats. Similarly, Pepe et al. [53] found that certain AMFs can maintain functional extraradical mycelial networks even after host plant senescence, suggesting a survival strategy that is decoupled from the host’s lifespan. In addition, Behm and Kiers [54] described high intraspecific trait plasticity in some AMFs, which may enable them to adjust growth, colonization, and sporulation in response to changing environmental conditions. Indicator species analysis supports these findings, revealing that the majority of significant indicator OTUs are specialized toward either root or hyphal fractions. Root-specific indicators, such as unassigned Glomeromycetes, and hyphal-specific indicators, including *Paraglomus* VTX00348 and *Glomus* VTX00219, reflected the distribution patterns observed in dominant taxa. These results reinforce the notion that propagule-type specialization is a widespread feature within AMF communities [14,55]. By contrast, fewer indicator taxa were associated with spore fractions, suggesting that sporulation may function primarily as a long-term survival and dispersal mechanism rather than an immediate colonization strategy. This interpretation is consistent with prior observations that spores persist under environmental stress but contribute less to rapid community assembly [1,13]. Taken together, these findings indicate that both dominant and indicator taxa exhibit distinct preferences for specific propagule types. Propagule identity, therefore, plays a critical role in shaping AMF community structure and functional dynamics across diverse soil environments. Additionally, recently increasing attention has been given to the role of biotic interactions, especially hyphosphere-associated microbes, in shaping AMF development. For instance, certain mycorrhiza helper bacteria can enhance spore germination and hyphal elongation by releasing signaling compounds or mobilizing nutrients [56,57]. These biotic interactions may also influence propagule-type success and should be considered in future studies investigating AMF colonization strategies. Further studies focusing on these microbial dynamics may deepen our understanding of the ecological mechanisms underlying propagule-specific adaptation and community assembly in AMF.

## 5. Conclusions

This study demonstrates that AMF taxa exhibit functional specialization based on propagule type, which strongly influences colonization strategies and early stage community assembly. Comprehensive profiling of AMF communities across root, hyphal, and spore fractions from three distinct inoculum sources revealed clear patterns. *Glomus* species were predominantly root-associated, *Paraglomus* taxa favored extraradical hyphal proliferation, and *Scutellospora* displayed generalist tendencies across all propagule types. These trends were further supported by indicator species analysis, which identified propagule type as a stronger determinant of AMF community structure than soil origin. Spore fractions harbored fewer specialist taxa, reinforcing their role in long-term persistence and dispersal rather than active colonization. Together, these findings contribute to a broader understanding of AMF life-history strategies and underscore the ecological significance of propagule identity in shaping fungal communities. Future research should investigate how environmental conditions, host identity, and temporal dynamics interact with propagule-driven processes to influence AMF community development across ecosystems. A deeper understanding of propagule ecology will be essential for advancing AMF-based applications in ecosystem restoration and sustainable agriculture.

## Data Availability

Raw sequence reads are deposited in the DNA Data Bank of Japan (DDBJ) under the accession number PRJDB20733. Sample metadata is also available in the DDBJ under the same accession number PRJDB20733.

## Supplementary Materials

The following supporting information can be downloaded at: https://www.mdpi.com/article/10.3390/microorganisms13061430/s1, Figure S1: Schematic overview of soil preparation, plant cultivation, propagule fractionation, and sequencing workflow. Figure S2: Venn diagram showing the distribution of AMF OTUs across the three soil types: Sandy regosol, andosol, and gleysol. Figure S3: Relative abundance of AMF genera detected in hyphae, root, and spore fractions across three soil types (sandy regosol, andosol, and gleysol) and three sampling time points (2, 4, and 6 weeks). Figure S4: Alpha diversity (Richness) of AMF communities across propagule types (hyphae, root, spore) in three soil inoculum sources—Sandy Regosol, Andosol, and Gleysol—at different sampling times: (a) 2 weeks, (b) 4 weeks, and (c) 6 weeks after transplanting. Table S1: List of 23 OTUs shared across all three soil types. Table S2: Taxonomic identity and dominant propagule fraction of the top 20 most abundant AMF OTUs across all samples. Table S3: Results of three-way ANOVA testing the effects of soil type, propagule type, and sampling week, and their interactions, on AMF alpha diversity (Richness). Table S4: Results of term-based PERMANOVA showing the individual and interactive effects of soil type, propagule fraction, and sampling week on AMF community composition based on weighted UniFrac distances. Table S5: List of OTUs significantly associated with propagule types based on indicator species analysis.

## Abbreviations

The following abbreviations are used in this manuscript:

AMF	Arbuscular Mycorrhizal Fungi
OTU	Operational Taxonomic Unit
DNA	Deoxyribonucleic Acid
PCR	Polymerase Chain Reaction
rRNA	Ribosomal Ribonucleic Acid
PCoA	Principal Coordinates Analysis
