# Supplementary material for: Propagule-Type Specificity in Arbuscular Mycorrhizal Fungal Communities in Early Growth of Allium tuberosum
Source: Microorganisms. 2025 Jun 19;13(6):1430. doi: 10.3390/microorganisms13061430 (PMC12196218; doi:10.3390/microorganisms13061430)
Supplement: Supplementary file 1 [file microorganisms-13-01430-s001.zip › microorganisms-3672998-supplementary.pdf]

Propagule-Type Specificity in Arbuscular Mycorrhizal Fungal Communities in Early Growth of *Allium tuberosum*

Irem Arslan[1], Kohei Takahashi[1], Naoki Harada[2], Kazuki Suzuki[2*]

[1] Graduate School of Science and Technology, Niigata University, 8050 Ikarashi-2, Nishi-ku, Niigata 950-2181, Japan

[2] Institute of Science and Technology, Niigata University, 8050 Ikarashi-2, Nishi-ku, Niigata 950-2181, Japan

[*]Corresponding author:
Kazuki Suzuki
Address: Institute of Science and Technology, Niigata University, 8050 Ikarashi-2, Nishi-ku, Niigata 950-2181, Japan
e-mail: suzukik@agr.niigata-u.ac.jp; Tel: +81-25-262-6687

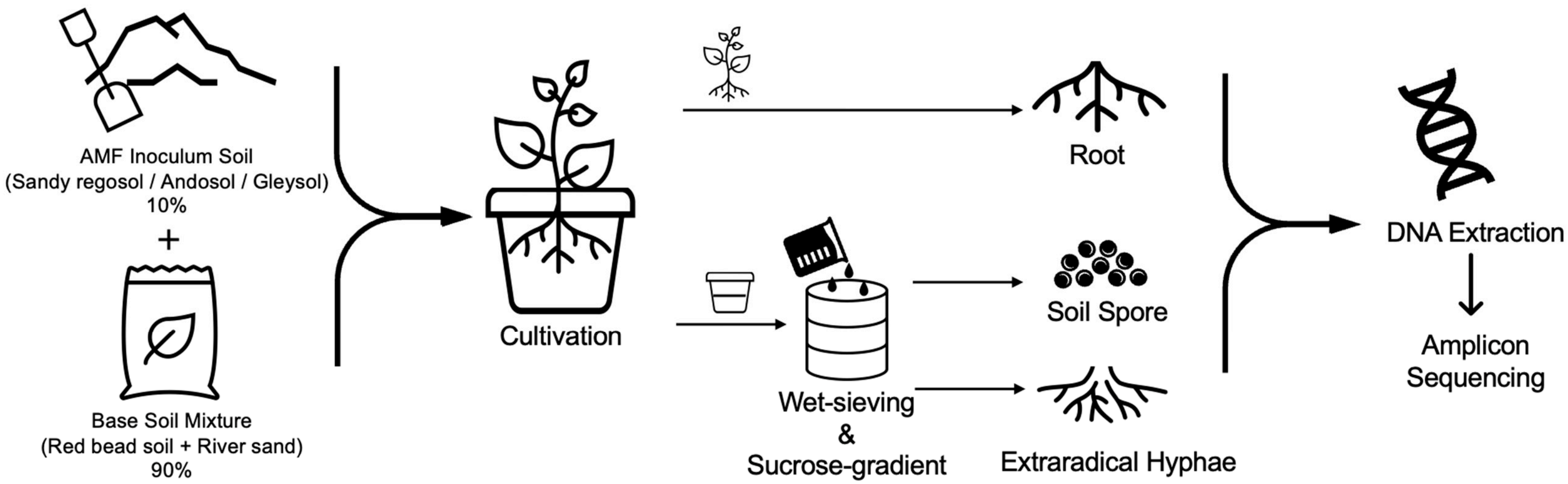

Supplementary Figure S1 Schematic overview of soil preparation, plant cultivation, propagule fractionation, and sequencing workflow

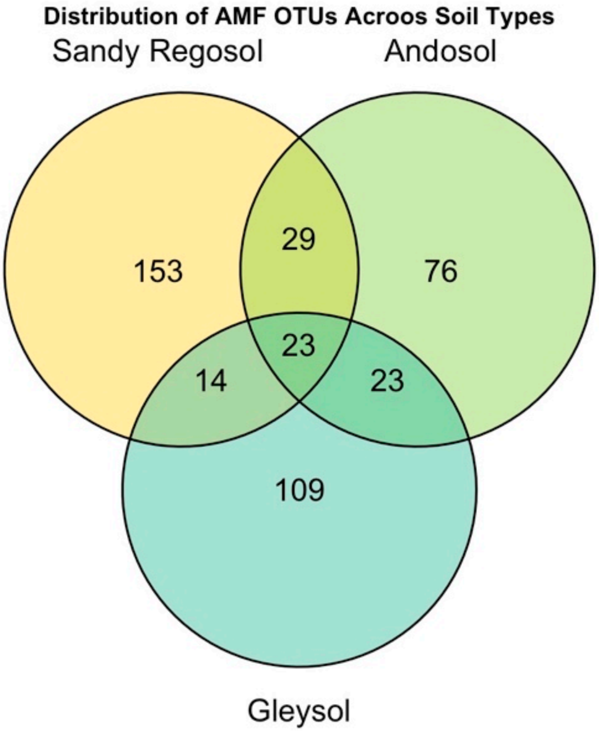

Supplementary Figure S2 Venn diagram showing the distribution of AMF OTUs across the three soil types: Sandy regosol, andosol, and gleysol.

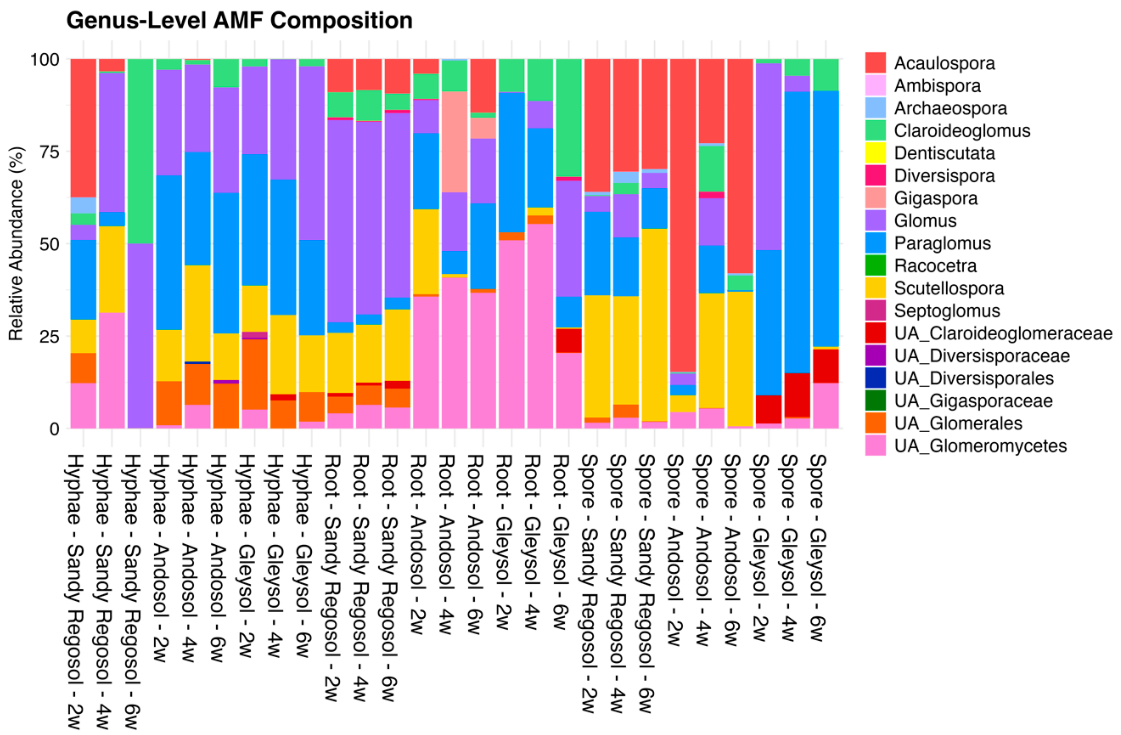

Supplementary Figure S3 Relative abundance of AMF genera detected in hyphae, root, and spore fractions across three soil types (sandy regosol, andosol, and gleysol) and three sampling time points (2, 4, and 6 weeks).

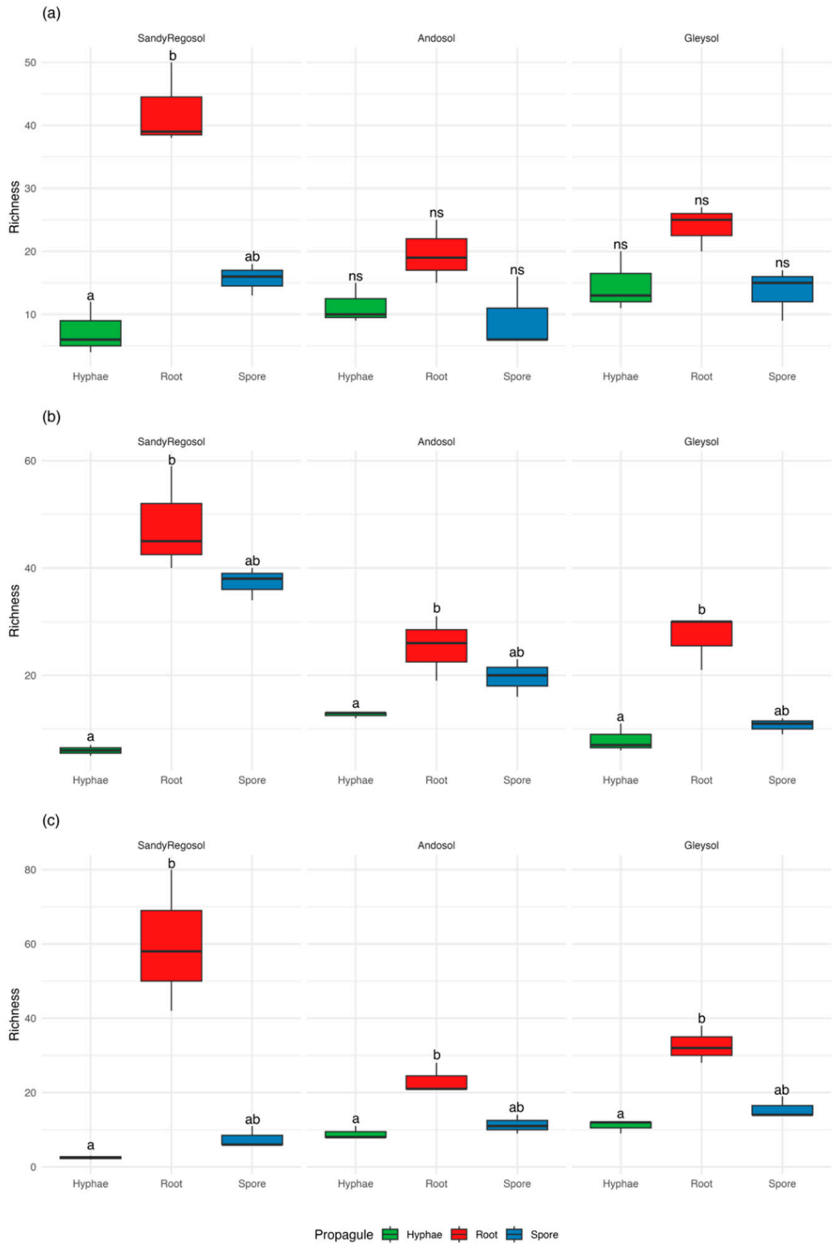

Supplementary Figure S4 Alpha diversity (Richness) of AMF communities across propagule types (hyphae, root, spore) in three soil inoculum sources—Sandy Regosol, Andosol, and Gleysol—at different sampling times: **(a)** 2 weeks, **(b)** 4 weeks, and **(c)** 6 weeks after transplanting. Richness values were calculated at the OTU level. Boxplots represent the median, interquartile range, and data spread for each propagule type within each soil. Statistical comparisons were conducted separately for each soil type using the Kruskal–Wallis test followed by Dunn's post hoc test with Benjamini–Hochberg correction. Different letters indicate statistically significant differences among propagule types within the same soil ($p < 0.05$). "ns" indicates no significant difference.

Supplementary Table S1 List of 23 OTUs shared across all three soil types. Taxonomic classification is shown at the species, genus, family, class and order levels based on sequence identity.

OTU	Species	Genus	Family	Order	Class	Phylum
OTU001	Unassigned *Scutellospora*	*Scutellospora*	Gigasporaceae	Diversisporales	Glomeromycetes	Mucoromycota
OTU003	VTX00348	*Paraglomus*	Paraglomeraceae	Paraglomerales	Paraglomeromycetes	Mucoromycota
OTU004	VTX00219	*Glomus*	Glomeraceae	Glomerales	Glomeromycetes	Mucoromycota
OTU005	Unassigned Glomeromycetes	Unassigned Glomeromycetes	Unassigned Glomeromycetes	Unassigned Glomeromycetes	Glomeromycetes	Mucoromycota
OTU006	Unassigned *Paraglomus*	*Paraglomus*	Paraglomeraceae	Paraglomerales	Paraglomeromycetes	Mucoromycota
OTU009	Unassigned *Glomus*	*Glomus*	Glomeraceae	Glomerales	Glomeromycetes	Mucoromycota
OTU010	VTX00279	*Claroideoglomus*	Claroideoglomeraceae	Glomerales	Glomeromycetes	Mucoromycota
OTU011	VTX00024	*Acaulospora*	Acaulosporaceae	Diversisporales	Glomeromycetes	Mucoromycota
OTU017	VTX00166	*Glomus*	Glomeraceae	Glomerales	Glomeromycetes	Mucoromycota
OTU018	Unassigned Glomerales	Unassigned Glomerales	Unassigned Glomerales	Glomerales	Glomeromycetes	Mucoromycota
OTU019	VTX00092	*Glomus*	Glomeraceae	Glomerales	Glomeromycetes	Mucoromycota
OTU020	VTX00308	*Paraglomus*	Paraglomeraceae	Paraglomerales	Paraglomeromycetes	Mucoromycota
OTU025	VTX00225	*Claroideoglomus*	Claroideoglomeraceae	Glomerales	Glomeromycetes	Mucoromycota
OTU026	VTX00175	*Glomus*	Glomeraceae	Glomerales	Glomeromycetes	Mucoromycota
OTU036	Unassigned Glomeromycetes	Unassigned Glomeromycetes	Unassigned Glomeromycetes	Unassigned Glomeromycetes	Glomeromycetes	Mucoromycota
OTU043	VTX00348	*Paraglomus*	Paraglomeraceae	Paraglomerales	Paraglomeromycetes	Mucoromycota
OTU054	VTX00214	*Glomus*	Glomeraceae	Glomerales	Glomeromycetes	Mucoromycota
OTU058	Unassigned Glomeromycetes	Unassigned Glomeromycetes	Unassigned Glomeromycetes	Unassigned Glomeromycetes	Glomeromycetes	Mucoromycota

OTU076	Unassigned Paraglomus	*Paraglomus*	Paraglomeraceae	Paraglomerales	Paraglomeromycetes	Mucoromycota
OTU113	Unassigned Glomeromycetes	Unassigned Glomeromycetes	Unassigned Glomeromycetes	Unassigned Glomeromycetes	Glomeromycetes	Mucoromycota
OTU115	VTX00279	*Claroideoglomus*	Claroideoglomeraceae	Glomerales	Glomeromycetes	Mucoromycota
OTU121	Unassigned Diversisporaceae	Unassigned Diversisporaceae	Diversisporaceae	Diversisporales	Glomeromycetes	Mucoromycota
OTU172	Unassigned Glomeromycetes	Unassigned Glomeromycetes	Unassigned Glomeromycetes	Unassigned Glomeromycetes	Glomeromycetes	Mucoromycota

Supplementary Table S2 Taxonomic identity and dominant propagule fraction of the top 20 most abundant AMF OTUs across all samples. OTUs were taxonomically classified at the genus and species level based on 18S rRNA sequencing, and their dominant propagule type (root, spore, or hyphae) was determined by relative abundance across fractions.

OTU	Species	Genus	Family	Order	Class	Phylum	Dominant Fraction
OTU001	Unassigned *Scutellospora*	*Scutellospora*	Gigasporaceae	Diversisporales	Glomeromycetes	Mucoromycota	Hyphae
OTU002	VTX00024	*Acaulospora*	Acaulosporaceae	Diversisporales	Glomeromycetes	Mucoromycota	Spore
OTU003	VTX00348	*Paraglomus*	Paraglomeraceae	Paraglomerales	Paraglomeromycetes	Mucoromycota	Hyphae
OTU004	VTX00219	*Glomus*	Glomeraceae	Glomerales	Glomeromycetes	Mucoromycota	Hyphae
OTU005	Unassigned Glomeromycetes	Unassigned Glomeromycetes	Unassigned Glomeromycetes	Unassigned Glomeromycetes	Glomeromycetes	Mucoromycota	Root
OTU006	Unassigned *Paraglomus*	*Paraglomus*	Paraglomeraceae	Paraglomerales	Paraglomeromycetes	Mucoromycota	Hyphae
OTU007	VTX00281	*Paraglomus*	Paraglomeraceae	Paraglomerales	Paraglomeromycetes	Mucoromycota	Spore
OTU010	VTX00279	*Claroideoglomus*	Claroideoglomeraceae	Glomerales	Glomeromycetes	Mucoromycota	Spore
OTU008	VTX00067	*Glomus*	Glomeraceae	Glomerales	Glomeromycetes	Mucoromycota	Hyphae
OTU009	Unassigned *Glomus*	*Glomus*	Glomeraceae	Glomerales	Glomeromycetes	Mucoromycota	Hyphae
OTU011	VTX00024	*Acaulospora*	Acaulosporaceae	Diversisporales	Glomeromycetes	Mucoromycota	Spore
OTU012	Unassigned Glomeromycetes	Unassigned Glomeromycetes	Unassigned Glomeromycetes	Unassigned Glomeromycetes	Glomeromycetes	Mucoromycota	Root
OTU013	VTX00308	*Paraglomus*	Paraglomeraceae	Paraglomerales	Paraglomeromycetes	Mucoromycota	Spore
OTU014	Unassigned Glomeromycetes	Unassigned Glomeromycetes	Unassigned Glomeromycetes	Unassigned Glomeromycetes	Glomeromycetes	Mucoromycota	Hyphae
OTU015	VTX00049	*Scutellospora*	Gigasporaceae	Diversisporales	Glomeromycetes	Mucoromycota	Spore
OTU016	Unassigned *Claroideoglomus*	Unassigned *Claroideoglomus*	Claroideoglomeraceae	Glomerales	Glomeromycetes	Mucoromycota	Spore

OTU017	VTX00166	Glomus	Glomeraceae	Glomerales	Glomeromycetes	Mucoromycota	Root
OTU018	Unassigned Glomerales	Unassigned Glomerales	Unassigned Glomerales	Glomerales	Glomeromycetes	Mucoromycota	Hyphae
OTU019	VTX00092	Glomus	Glomeraceae	Glomerales	Glomeromycetes	Mucoromycota	Hyphae
OTU026	VTX00175	Glomus	Glomeraceae	Glomerales	Glomeromycetes	Mucoromycota	Hyphae

Supplementary Table S3 Results of three-way ANOVA testing the effects of soil type, propagule type, and sampling week, and their interactions, on AMF alpha diversity (Richness). Significant interactions were detected between soil type and propagule type ($p = 0.0007$) and among soil type, propagule type, and sampling week ($p = 0.0034$), whereas individual main effects were not statistically significant.

Effect	Sum of Squares	df	F-value	p-value	Significance
Soil	80.89	2	1.31	0.2780	ns
Propagule	180.22	2	2.92	0.0626	.
Week	20.67	2	0.34	0.7168	ns
Soil × Propagule	699.78	4	5.67	0.0007	***
Soil × Week	63.71	4	0.52	0.7239	ns
Propagule × Week	81.26	4	0.66	0.6234	ns
Soil × Propagule × Week	833.00	8	3.38	0.0034	**
Residuals	1634.50	53			

Supplementary Table S4 Results of term-based PERMANOVA showing the individual and interactive effects of soil type, propagule fraction, and sampling week on AMF community composition based on weighted UniFrac distances. Statistical significance was determined using 999 permutations.

**Term**	**df**	**Sum of Squares**	**R^2**	**F-value**	**p-value**	**Significance**
Soil	2	1.3727	0.17900	14.8459	0.001	***
Propagule	2	1.0989	0.14330	11.8850	0.001	***
Week	2	0.1540	0.02008	1.6650	0.096	.
Soil × Propagule	4	1.3666	0.17821	7.3901	0.001	***
Soil × Week	4	0.2065	0.02693	1.1166	0.307	ns
Propagule × Week	4	0.3095	0.04036	1.6736	0.054	.
Soil × Propagule × Week	8	0.7101	0.09259	1.9198	0.004	**
Residual	53	2.4503	0.31952			
Total	79	7.6686	1.00000			

Supplementary Table S5 List of OTUs significantly associated with propagule types based on indicator species analysis. The table includes OTU IDs, taxonomic assignments, associated propagule types (hyphae, root, spore, or combinations thereof), indicator values (IndVal), and adjusted p-values (Benjamini–Hochberg correction). OTUs with adjusted p-values < 0.05 were considered significant indicators. Taxa are sorted according to their associated propagule type and indicator strength. This analysis was conducted separately across soil types and then merged based on consistent indicator patterns.

OTU	Species	Genus	Family	Order	Class	Phylum
OTU003	VTX00348	*Paraglomus*	Paraglomeraceae	Paraglomerales	Paraglomeromycetes	Mucoromycota
OTU004	VTX00219	*Glomus*	Glomeraceae	Glomerales	Glomeromycetes	Mucoromycota
OTU005	Unassigned Glomeromycetes	Unassigned Glomeromycetes	Unassigned Glomeromycetes	Unassigned Glomeromycetes	Glomeromycetes	Mucoromycota
OTU009	Unassigned *Glomus*	*Glomus*	Glomeraceae	Glomerales	Glomeromycetes	Mucoromycota
OTU011	VTX00024	*Acaulospora*	Acaulosporaceae	Diversisporales	Glomeromycetes	Mucoromycota
OTU018	Unassigned Glomerales	Unassigned Glomerales	Unassigned Glomerales	Glomerales	Glomeromycetes	Mucoromycota
OTU019	VTX00092	*Glomus*	Glomeraceae	Glomerales	Glomeromycetes	Mucoromycota
OTU022	Unassigned Glomerales	Unassigned Glomerales	Unassigned Glomerales	Glomerales	Glomeromycetes	Mucoromycota
OTU029	VTX00348	*Paraglomus*	Paraglomeraceae	Paraglomerales	Paraglomeromycetes	Mucoromycota
OTU033	Unassigned *Glomus*	*Glomus*	Glomeraceae	Glomerales	Glomeromycetes	Mucoromycota
OTU034	VTX00100	*Glomus*	Glomeraceae	Glomerales	Glomeromycetes	Mucoromycota
OTU038	Unassigned Glomeromycetes	Unassigned Glomeromycetes	Unassigned Glomeromycetes	Unassigned Glomeromycetes	Glomeromycetes	Mucoromycota
OTU040	VTX00352	*Paraglomus*	Paraglomeraceae	Paraglomerales	Paraglomeromycetes	Mucoromycota
OTU041	Unassigned Paraglomus	*Paraglomus*	Paraglomeraceae	Paraglomerales	Paraglomeromycetes	Mucoromycota
OTU066	Unassigned Glomeromycetes	Unassigned Glomeromycetes	Unassigned Glomeromycetes	Glomerales	Glomeromycetes	Mucoromycota

OTU074	Unassigned *Paraglomus*	*Paraglomus*	Paraglomeraceae	Paraglomerales	Paraglomeromycetes	Mucoromycota
OTU085	Unassigned *Glomus*	*Glomus*	Glomeraceae	Glomerales	Glomeromycetes	Mucoromycota
OTU110	Unassigned Glomeromycetes	Unassigned Glomeromycetes	Unassigned Glomeromycetes	Unassigned Glomeromycetes	Glomeromycetes	Mucoromycota
OTU115	VTX00279	*Claroideoglomus*	Claroideoglomeraceae	Glomerales	Glomeromycetes	Mucoromycota
OTU117	VTX00348	*Paraglomus*	Paraglomeraceae	Paraglomerales	Paraglomeromycetes	Mucoromycota
OTU119	VTX00308	*Paraglomus*	Paraglomeraceae	Paraglomerales	Paraglomeromycetes	Mucoromycota
